# Long non-coding RNA CASC9 promotes tumor growth and metastasis via modulating FZD6/Wnt/β-catenin signaling pathway in bladder cancer

**DOI:** 10.1186/s13046-020-01624-9

**Published:** 2020-07-16

**Authors:** Yonghao Zhan, Lianghao Zhang, Shuanbao Yu, Jianguo Wen, Yuchen Liu, Xuepei Zhang

**Affiliations:** 1 Department of Urology, The First Affiliated Hospital of Zhengzhou University, No. 1 Jianshe East Road, Zhengzhou, 450052 China; 2 Key Laboratory of Precision Diagnosis and Treatment for Chronic Kidney Disease in Henan Province, No. 1 Jianshe East Road, Zhengzhou, 450052 China; 3 Shenzhen Second People’s Hospital, The First Affiliated Hospital of Shenzhen University, Health Science Center, Shenzhen, 518035 China

**Keywords:** Bladder cancer, CASC9, FZD6, lncRNA, miR-497-5p

## Abstract

**Background:**

Accumulating evidence have highlighted the importance of long noncoding RNAs (lncRNAs) in multiple cancers development and progression. Cancer susceptibility candidate 9 (CASC9) is a novel long non-coding RNA and plays important regulatory role in diverse biological processes of cancers. However, the clinical significance and molecular mechanism of CASC9 in bladder cancer is still unknown.

**Methods:**

Comprehensive lncRNAs profiling analysis were conducted to identify lncRNAs profile alterations and uncover valuable lncRNA candidates for bladder cancer. The expression level of CASC9 was determined in a total of 106 patients with bladder cancer. Loss-of-function experiments were performed to identify the functions of CASC9 in tumor growth and metastasis of bladder cancer in vitro and in vivo. Bioinformatics analysis and further experiments were performed to explore the molecular mechanisms underlying the functions of CASC9.

**Results:**

This study found that CASC9 expression was markedly upregulated in bladder cancer and related to histological grade, TNM stage and prognosis. Knockdown of CASC9 inhibited tumor growth and metastasis of bladder cancer in vitro and in vivo. Mechanistically, we found that CASC9 functions as a miRNA sponge to positively regulate FZD6 expression and subsequently activates Wnt/β-catenin signaling pathway, thus playing an oncogenic role in bladder cancer pathogenesis.

**Conclusion:**

In summary, lncRNA CASC9 plays a critical regulatory role in bladder cancer. The CASC9/miR-497-5p/ FZD6 axis provides insights for regulatory mechanism of bladder cancer, and new strategies for clinical practice.

## Background

Bladder cancer (BC) is the sixth most frequent malignancy in males and represents one of the most common genitourinary malignancies all over the world [1, 2]. The cause of BC is still unknown and the prognosis of patients with BC is poor under conventional treatments [3, 4]. Thus, the effective prognostic and therapeutic targets for BC are urgently needed.

The long non-coding RNAs (lncRNAs) are important new members of the ncRNA family with greater than 200 nucleotides in length, and lacking of protein coding probability [5, 6]. Over the past decades, the rapid development of RNA genomics has highlighted the important role of lncRNAs in many human diseases, especially in cancers [7–10]. Recently, a growing number of evidence has showed that many lncRNAs play important regulatory roles in diverse biological processes of BC, such as BLACAT2, LNMAT1, LNMAT2, PTENP1, UCA-1, HOTAIR, and etc. [11–18]. Cancer susceptibility candidate 9 (CASC9, Gene ID 101805492 in NCBI records) is a novel identified lncRNA located at 8q21.11 [19]. Recently, CASC9 originally was identified as a potential biomarker and was involved in the development and progression of multiple cancers [20–25]. For example, CASC9 forms a complex with HNRNPL and co-regulates target genes expression and subsequently activates AKT-signaling pathway, in hepatocellular carcinoma. CASC9 activates LAMC2 expression through altering LAMC2 promoter H3K27me3 level by recruits CBP and subsequently promotes esophageal squamous cell carcinoma metastasis. CASC9 also recruiting EZH2 to PDCD4 promoter and alters H3K27me3 level and subsequently promotes esophageal squamous cell carcinoma metastasis by activating PDCD4 expression [24]. However, the biological function and underlying mechanism of action of CASC9 in BC is completely unknown.

In the present study, we performed comprehensive lncRNAs profiling analyses to identify lncRNAs profile alterations and uncover valuable lncRNA candidates for BC using The Cancer Genome Atlas (TCGA-BLCA) data and Gene Expression Omnibus (GEO) data. We found that CASC9 was significantly up-regulated in BC and positively correlated with poor prognosis. Meanwhile, CASC9 expression was markedly upregulated in BC and related to histological grade, TNM stage and prognosis. Furthermore, further experiments demonstrated that knockdown of CASC9 inhibited growth and metastasis of bladder cancer cells (BCCs) in vitro and in vivo. Mechanistically, we found CASC9 was mainly distributed in the cytoplasm and knockdown of CASC9 increased miR-497-5p expression and subsequently inhibited FZD6 expression, in a ceRNA-dependent way. Moreover, knockdown of miR-497-5p reversed FZD6 expression inhibition and reversed malignant phenotypes inhibition of BCCs. Together, our results suggest that CASC9 is a powerful tumor biomarker, which highlight its potential clinical utility as a promising diagnostic and therapeutic target of BC.

## Materials and methods

### Data extraction and bioinformatics analysis

Gene expression profiles was downloaded from The Cancer Genome Atlas (TCGA-BLCA) database and the Gene Expression Omnibus (GSE89006) database. The edgeR package was used to analysis differentially expressed lncRNA in TCGA-BLCA detasets. The limma package was used to analysis differentially expressed lncRNA in GEO detasets. The GEPIA (http://gepia.cancer-pku.cn/index.html) was used to analysis prognosis of BC in TCGA-BLCA detasets. The lncLocator was used to predict the subcellular localization of CASC9.

### Clinical sample collection and cell culture

Clinical sample collection and cell culture were performed following standard protocols as previously reported [18]. Clinicopathological features of patients was shown in Supplementary Table 1. The normal urothelial cell line and BC cell lines were purchased from the Institute of Cell Research, Chinese Academy of Sciences, Shanghai, China.

### Quantitative real-time PCR and western blotting analysis

Quantitative real-time PCR and western blotting analysis were performed following standard protocols as previously reported [18]. The detailed primer sequences included in this study are shown in Supplementary Table 2. The detailed protocols included in this study are also shown in Supplementary materials and methods. The primary antibody FZD6/Ki67/MMP14 and secondary antibody were purchased from Abcam, Hong Kong, China. The primary antibody β-catenin/E-cadherin/N-cadherin/vimentin/slug were purchased from Cell Signaling Technology, USA.

### Cell proliferation and cell metastasis assays

Cell Counting Kit-8 (CCK-8) and ethynyl-2-deoxyuridine (EdU)-incorporation assay were used to determine BC cell proliferation. Wound-healing assays and transwell assays were used to determine the migratory abilities and invasive abilities of BCCs, respectively. Cell Counting Kit-8 (CCK-8), ethynyl-2-deoxyuridine (EdU)-incorporation assay, wound-healing assays and transwell assays were performed following standard protocols as previously reported [16]. The detailed protocols included in this study are also shown in Supplementary materials and methods.

### Mouse model experiments

All animal experiments were approved by the Institutional Animal Care and Use Committee (IACUC) of The First Affiliated Hospital of Zhengzhou University and conducted in accordance with its recommendations and ethical regulations. For the tumour xenograft implantation experiment, 1 × 10^6^ SW780 cells were injected subcutaneously into 5-week-old male BALB/c nude mice (Vital River, Beijing, China), which were subsequently sacrificed 5 weeks later. For the metastasis experiment, 1 × 10^5^ 5637-Luc cells were suspended in 200 μL PBS and injected into the lateral tail veins of 5-week-old male B-NDG mice (BIOCYTOGEN, Beijing, China). Four weeks later, mice were anaesthetized with isoflurane and D-luciferin sodium salt (150 mg/kg) was injected intraperitoneally, and cancer cells were detected with an in vivo imaging system, Xenogen IVIS (PerkinElmer, MA, USA). The total flux in photons per second was calculated and reported for each mouse’s lung and liver region using Living Image 4.3.1 (PerkinElmer/Caliper).

### FISH, immunohistochemistry and immunofluorescence

RNA fluorescent in situ hybridization (FISH), immunohistochemistry and immunostaining were performed following standard protocols as previously reported [16]. CASC9 and U6 probes were designed and synthesized by Ribobio Company and labeled with Cy3 fluorescent dye. The primary antibody FZD6/Ki67 were purchased from Abcam, Hong Kong, China. The primary antibody E-cadherin/N-cadherin and fluorescence secondary antibody were purchased from Cell Signaling Technology, USA.

### Statistical analyses

Quantitative data from three independent experiments were analyzed by Student’s t-test or χ2 test and results were expressed as mean ± SD. Kaplan-Meier survival analysis was used to evaluate the cumulative survival probability. *P* < 0.05 were considered statistically significant. All statistical tests were conducted using SPSS 19.0 (SPSS Inc. Chicago, IL, USA).

## Results

### CASC9 expression is up-regulated in BC

**Fig. 1:**
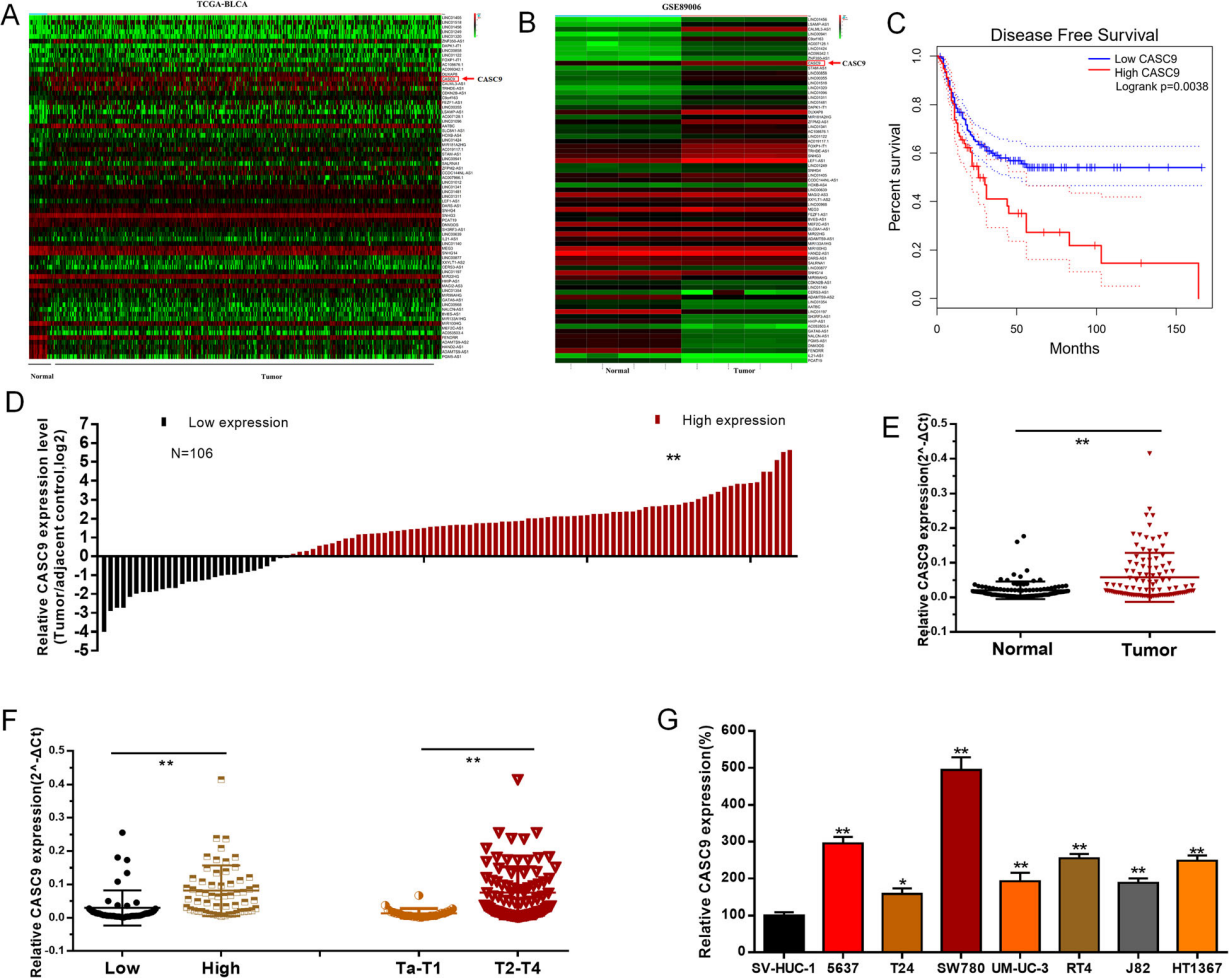
CASC9 expression is up-regulated in BC. **a** The most valuable lncRNA candidates in TCGA dataset were showed in the thermograph included CASC9. **b** The most valuable lncRNA candidates in GSE89006 datasets were showed in the thermograph included CASC9. **c** Higher CASC9 expression is related to BC patients’ shorter DFS in TCGA-BLCA dataset. **d** and **e** CASC9 expression is up-regulated in BC tissues compared with corresponding non-tumor tissues. **f** CASC9 expression is up-regulated in BC patients with advanced T stage and higher histological grade. **g** CASC9 expression is up-regulated in BC cell lines compared to normal urothelial cell line. Data are shown as mean ± SD. *P < 0.05; **P < 0.01

We conducted comprehensive lncRNAs expression profile analyses using TCGA-BLCA dataset and a GEO detaset (GSE89006) to identify lncRNAs profile alterations and uncover valuable lncRNA candidates for BC and explored their clinical relevance. We found CASC9 expression were up-regulated in TCGA-BLCA (Fig. 1a and Fig. S1A) and GSE89006 (Fig. 1b and Fig. S1B) datasets and higher CASC9 expression was associated with BC patients’ shorter DFS (Fig. 1c) in TCGA-BLCA dataset. The relative expression level of CASC9 was determined by qRT-PCR in BC cohort data to confirm the result of bioinformatics analysis. Meanwhile, CASC9 expression was up-regulated in BC tissues compared to paired non-tumor tissues (Fig. 1d and e) and higher CASC9 expression was positively correlated with advanced T stage and higher histological grade (Fig. 1f). Moreover, CASC9 expression was elevated in BC cell lines compared to SV-HUC-1 (Fig. 1g). The CASC9 specific shRNAs markedly down-regulated CASC9 expression in BC cells (Fig. S1C) and the CASC9 vector significantly increased CASC9 expression in BC cells (Fig. S1D). Correlation between CASC9 expression and clinicopathological features of BC patients was shown in Table 1.

**Table 1:** Correlation between CASC9 expression and clinicopathological features of UCB patients

Parameters Total	Group	Total	CASC9 expression		*P* value
			High	Low	
Gender	Male	79 (75%)	58 (55%)	21 (20%)	0.759
	Female	27 (25%)	19 (18%)	8 (8%)	
Age (years)	< 60	37 (35%)	27 (25%)	10 (9%)	0.956
	≥ 60	69 (65%)	50 (47%)	19 (18%)	
Tumor size (cm)	< 3 cm	42 (40%)	27 (25%)	15 (14%)	0.118
	≥ 3 cm	64 (60%)	50 (47%)	14 (13%)	
Multiplicity	Single	59 (56%)	38 (36%)	21 (20%)	0.033 *
	Multiple	47 (44%)	39 (37%)	8 (8%)	
Histological grade	L	48 (45%)	25 (24%)	23 (22%)	0.001 **
	H	58 (55%)	52 (49%)	6 (6%)	
Tumor stage T	Ta,T1	26 (25%)	10 (9%)	16 (15%)	0.001 **
	T2-T4	80 (75%)	67 (63%)	13 (12%)	
Lymph nodes metastasis	NO	92 (87%)	65 (61%)	27 (25%)	0.153
	YES	14 (13%)	13 (12%)	1 (1%)	

*P < 0.05; **P < 0.01. *P* < 0.05 was considered significant (Chi-square test between 2 groups)

### CASC9 promotes the proliferation of bladder cancer cells

**Fig. 2:**
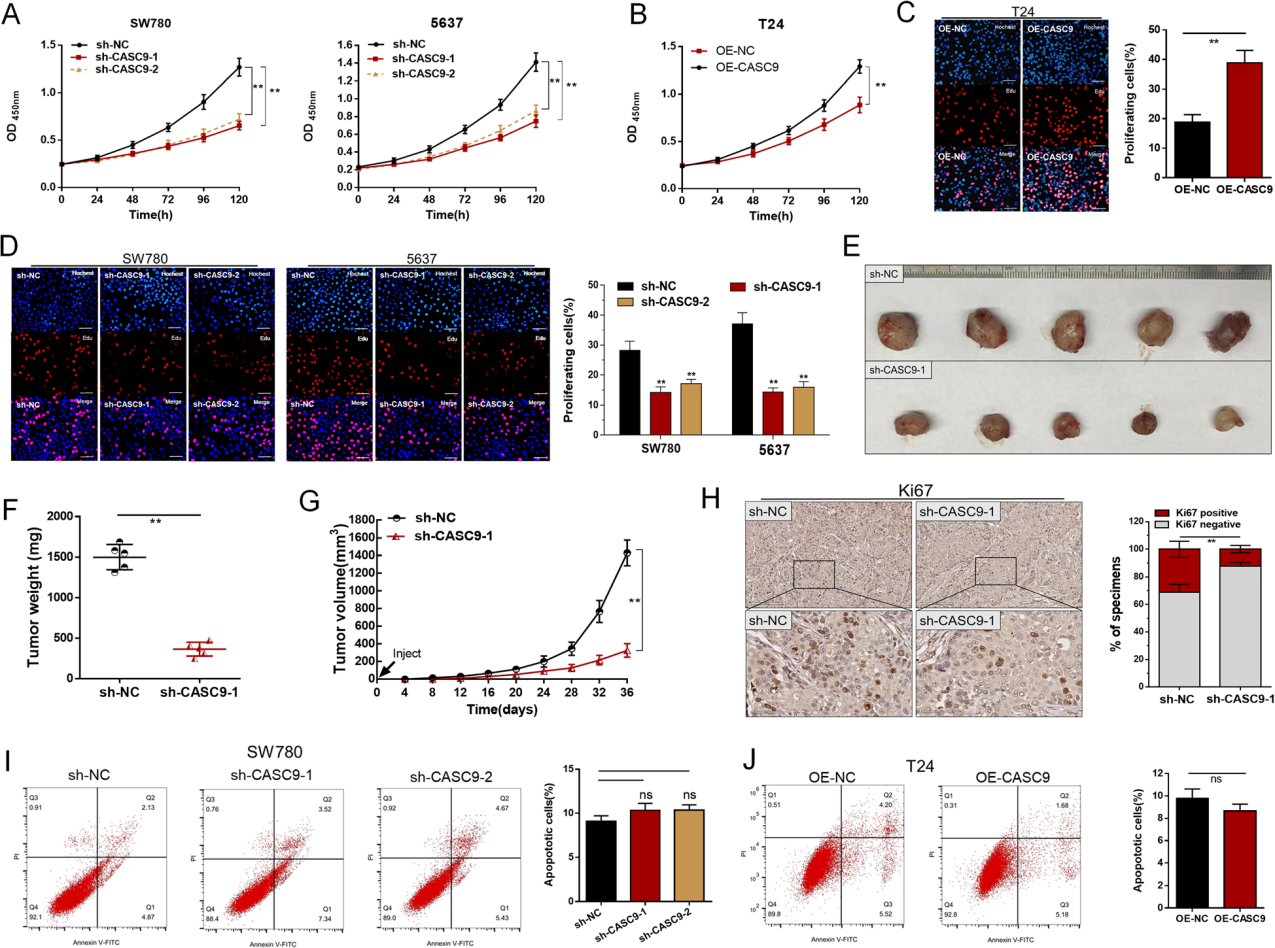
CASC9 promotes the proliferation but does not affect the apoptosis of BCCs. **a** and **b** The proliferation changes of BCCs were determined using CCK-8 assay in vitro. **c** and **d** The proliferation changes of BCCs were determined using Edu assay in vitro. **e** Tumours collected from mice are shown. **f** Tumor weight of shRNA-NC group was greater than that in the shRNA-CASC9 group. **g** Tumor growth of shRNA-NC was faster than that in the shRNA-CASC9 group. **h** The expression of Ki67 was determined using Immunohistochemistry. **i** and **j** The changes in bladder cancer cell apoptosis were determined using flow cytometry. The data are shown as the mean ± SD. *P < 0.05; **P < 0.01

To investigate whether CASC9 regulates cell proliferation of BC, we conducted CCK-8 assay and Edu assay to determine the proliferation changes of BCCs. Our results showed that knockdown of CASC9 inhibited the proliferation of BCCs (Fig. 2a and d) and overexpressing CASC9 promoted the proliferation of BCCs (Fig. 2b and c) in vitro. Meanwhile, we performed generation of xenograft to investigate the biological function and regulatory mechanism of CASC9 in vivo. Our result showed knockdown of CASC9 decreased tumor growth and tumor weight (Fig. 2e-g), and increased tumor free survival time (Fig. S2A). Moreover, we found knockdown of CASC9 decreased Ki67 expression (Fig. 2h) of BCCs in vivo. These results indicated that CASC9 promotes the proliferation of bladder cancer cells.

### CASC9 does not affect the apoptosis of bladder cancer cells

We further determined whether CASC9 regulates cell apoptosis of BCCs. The changes in bladder cancer cell apoptosis were determined using flow cytometry. Regrettably, there was no difference in the apoptosis of BCCs transfected with the corresponding specific shRNA (Fig. 2i) and overexpression vector (Fig. 2j). The results indicated that CASC9 does not affect the apoptosis of bladder cancer cells.

### CASC9 promotes the migration and invasion of bladder cancer cells

**Fig. 3:**
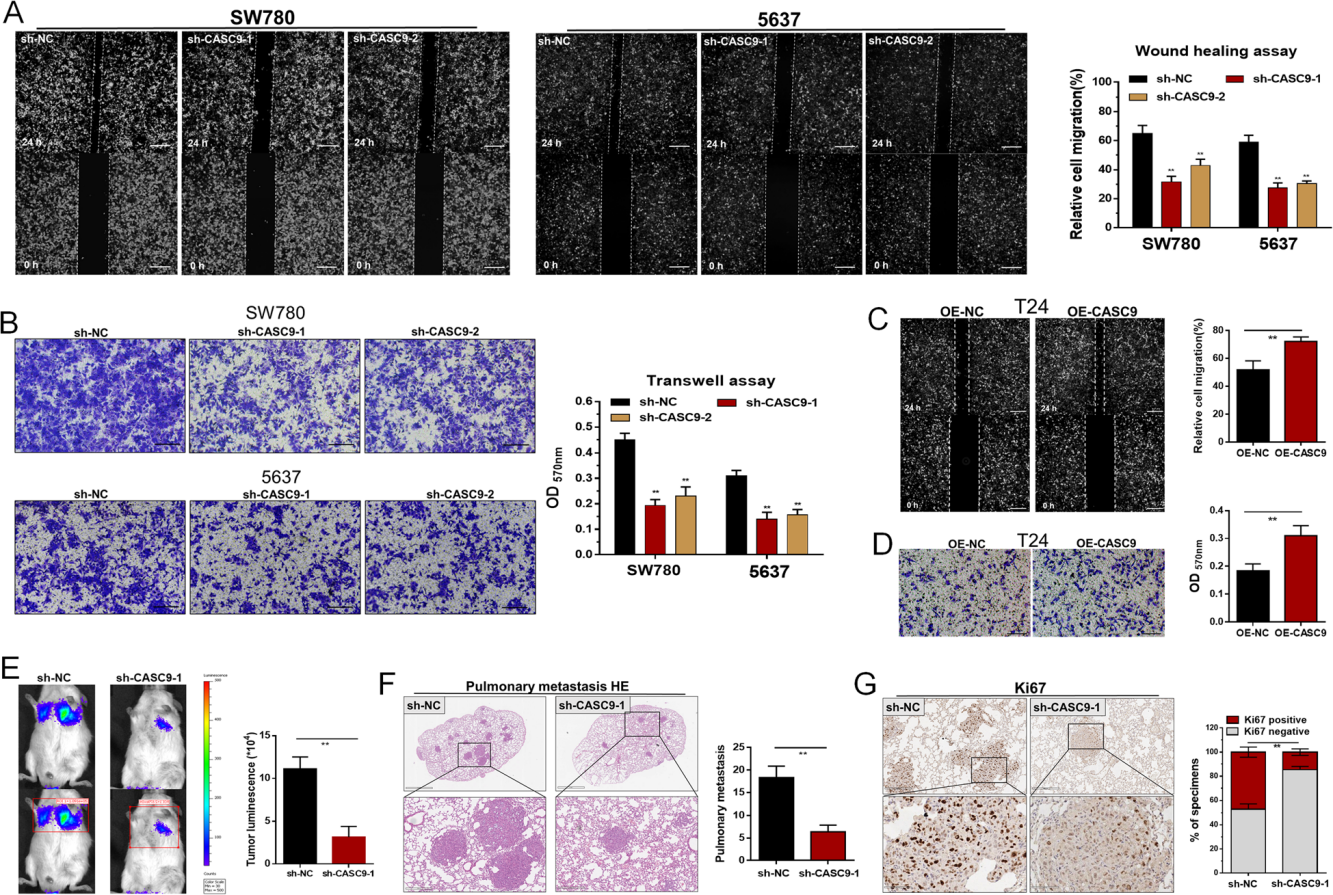
CASC9 promotes cell metastasis of BCCs in vitro and in vivo. **a** and **c** The migratory abilities of BCCs were determined using wound healing assay in vitro. **b** and **d** The invasive abilities of BCCs were determined using transwell assay in vitro. **e** The metastasis of BCCs we also determined using whole-body fluorescent imaging system, and knockdown of CASC9 decreased luciferase signals. **f** Knockdown of CASC9 significantly reduced the number of pulmonary metastases and metastases size. **g** Knockdown of CASC9 decreased Ki67 expression of BCCs in vivo. The data are shown as the mean ± SD. *P < 0.05; **P < 0.01

We further determined whether CASC9 regulates the migration and invasion of BCCs using wound healing assay and transwell assay, respectively. We found that knockdown of CASC9 inhibited migration (Fig. 3a) and invasion of BCCs (Fig. 3b). Meanwhile, we found overexpressing CASC9 promoted migration (Fig. 3c) and invasion (Fig. 3d) of BCCs. To investigate the biological function and regulatory mechanism of CASC9 in vivo, we also performed whole-body fluorescent imaging system. Our result showed knockdown of CASC9 decreased luciferase signals (Fig. 3e) but had no effect on mouse weight (Fig. S2C). The hematoxylin-eosin staining was performed to the lung tissue to observed the metastases in the groups (Fig. 3f), respectively. Our results showed knockdown of CASC9 significantly reduced the number of pulmonary metastases and metastases size (Fig. 3f). Moreover, further results showed knockdown of CASC9 decreased Ki67 (Fig. 3g) expression of BCCs in vivo. These results indicated that CASC9 promotes the migration and invasion of bladder cancer cells.

### CASC9 positively regulates the expression of FZD6

**Fig. 4:**
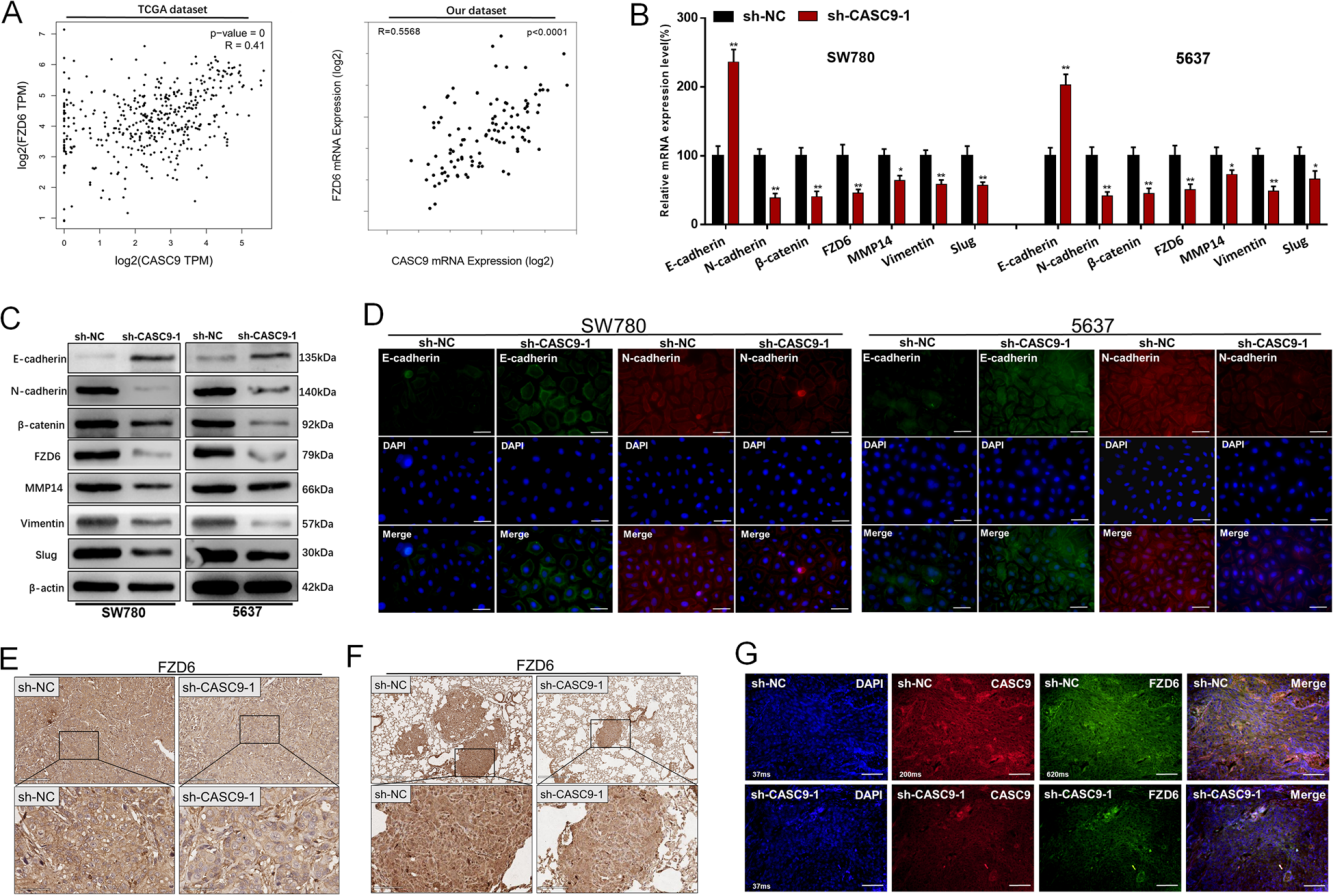
CASC9 positively regulates the expression of FZD6. **a** CASC9 expression was statistically positively correlated with FZD6 expression in BC. **b** The expression of FZD6/wnt/β-catenin markers were determined using qRT-PCR. **c** The expression of FZD6/wnt/β-catenin markers were determined using western blotting. **d** The expression of EMT markers were determined using immunofluorescence. **e** The expression of FZD6 in xenografts were determined using Immunohistochemistry. **f** The expression of FZD6 in pulmonary metastases were determined using Immunohistochemistry. **g** The expression of CASC9 and FZD6 were determined using FISH and immunofluorescence, respectively. Data are shown as mean ± SD. *P < 0.05; **P < 0.01

Through comprehensive transcriptional analysis using TCGA dataset and our dataset, we found that CASC9 expression were positively correlated with FZD6 expression in BC (Fig. 4a). Furthermore, FZD6 expression are up-regulated in BC (Fig. S3A) and FZD6 expression is related to the OS and DFS of BC patients in TCGA-BLCA dataset (Fig. S3B). Correlation between FZD6 expression and clinicopathological features of BC patients was shown in Table 2. Our result showed knockdown of CASC9 decreased FZD6, β-catenin expression and inhibited EMT of BCCs (Fig. 4b-d) in vitro. Meanwhile, we found knockdown of CASC9 decreased FZD6 (Fig. 4e-g) and inhibited EMT (Fig. S2B and D) of bladder cancer cells in vivo. Moreover, CASC9 and FZD6 were co-expressed in bladder cancer cells (Fig. 4g). These results indicated that CASC9 may activates Wnt/β-catenin signaling pathway via regulating FZD6 expression and subsequently promotes growth and metastasis of BCCs.

**Table 2:** Correlation between FZD6 expression and clinicopathological features of UCB patients

Parameters Total	Group	Total	FZD6 expression		*P* value
			High	Low	
Gender	Male	79 (75%)	56 (53%)	23 (22%)	0.682
	Female	27 (25%)	18 (17%)	9 (8%)	
Age (years)	< 60	37 (35%)	25 (24%)	12 (11%)	0.713
	≥ 60	69 (65%)	49 (46%)	20 (19%)	
Tumor size (cm)	< 3 cm	42 (40%)	24 (23%)	18 (17%)	0.021 *
	≥ 3 cm	64 (60%)	50 (47%)	14 (13%)	
Multiplicity	Single	59 (56%)	40 (38%)	19 (18%)	0.613
	Multiple	47 (44%)	34 (32%)	13 (12%)	
Histological grade	L	48 (45%)	26 (25%)	22 (21%)	0.003 **
	H	58 (55%)	48 (45%)	10 (9%)	
Tumor stage T	Ta,T1	26 (25%)	11 (10%)	15 (14%)	0.001 **
	T2-T4	80 (75%)	63 (59%)	17 (16%)	
Lymph nodes metastasis	NO	92 (87%)	63 (59%)	29 (27%)	0.650
	YES	14 (13%)	11 (10%)	3 (3%)	

*P < 0.05; **P < 0.01. *P* < 0.05 was considered significant (Chi-square test between 2 groups)

**Fig. 5:**
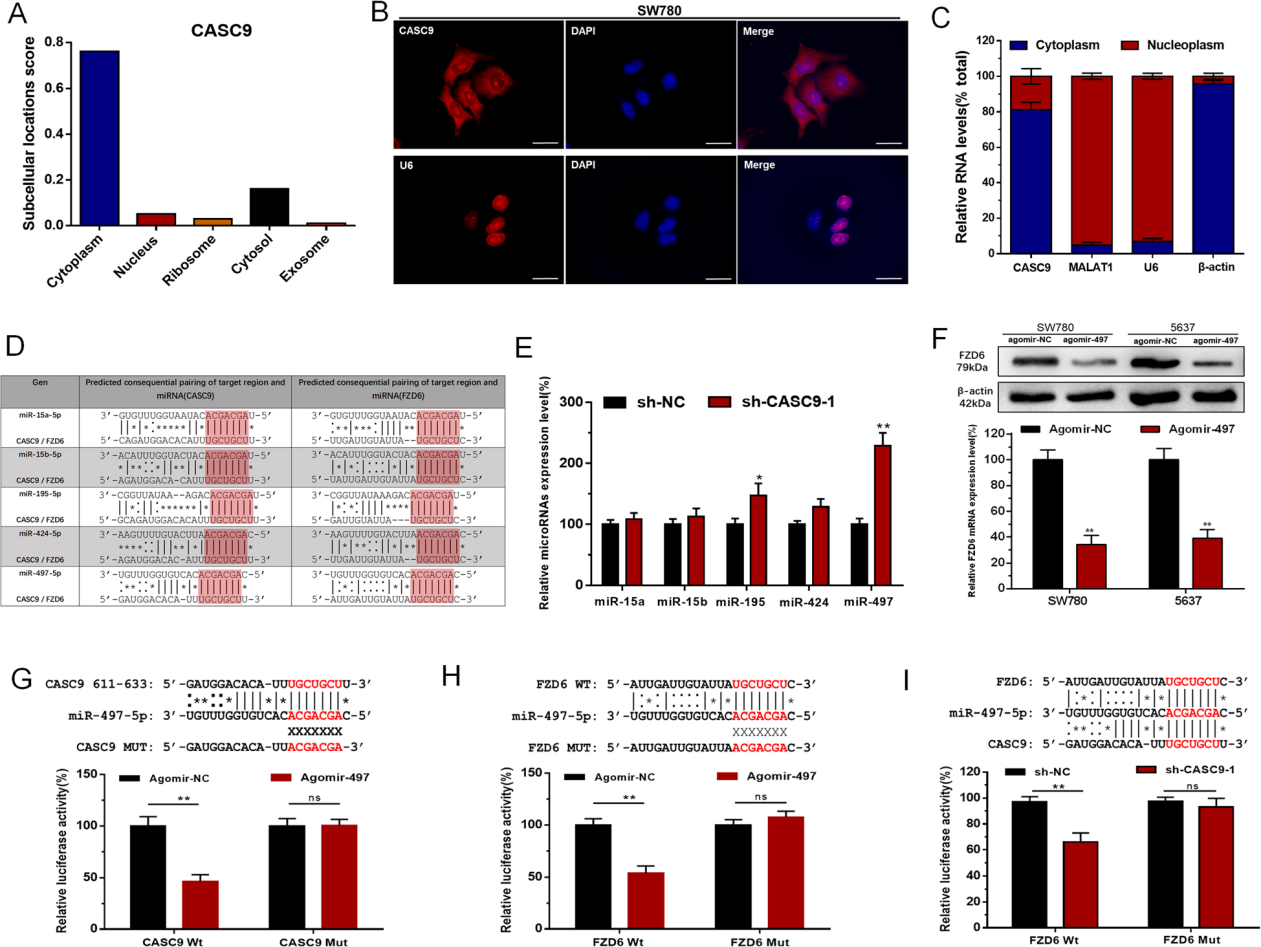
CASC9 positively regulates FZD6 expression via sponging miR-497-5p. **a** lncLocator results revealed that CASC9 was distributed mostly in the cytoplasm. **b** The RNA-FISH results revealed that CASC9 was distributed mostly in the cytoplasm of BCCs. **c** Subcellular localization of CASC9 and control genes analyzed with quantitative RT-PCR in biochemically fractionated SW780 cells. **d** The bio-information analysis results showed CASC9 and FZD6 have common putative binding sites with miR-497-5p cluster. **e** Knockdown of CASC9 increased miR-497-5p expression in BCCs. **f** Overexpressing miR-497-5p decreased FZD6 expression in BCCs. **g** CASC9 have putative binding sites with miR-497-5p and agomir-497 significantly inhibited luciferase activity of CASC9-Wt group. **h** The 3’UTR sequence of FZD6 is complementary to the seed sequence of miR-497-5p and agomir-497 significantly inhibited luciferase activity of FZD6-Wt group. **i** Knockdown of CASC9 decreased the luciferase activity of BCCs transfected with FZD6-Wt. Data are shown as mean ± SD. *P < 0.05; **P < 0.01

### CASC9 regulates FZD6 expression via sponging miR-497-5p

To explore the regulatory mechanism of CASC9 on FZD6, we predicted the subcellular localization of CASC9 using lncLocator (Fig. 5a), and performed RNA-FISH (Fig. 5b) and qRT-PCR (Fig. 5c) to verify the result in BCCs. The results revealed that CASC9 was distributed mostly in cytoplasm of BCCs. To elucidate whether CASC9 functioned as a ceRNA in BCCs, we used RegRNA 2.0 and Targetsacn 7.1 to predict potential shared target microRNA of CASC9 and FZD6. The results revealed that CASC9 and FZD6 have shared putative binding sites with miR-497-5p cluster (Fig. 5d). Furthermore, further experimental results showed knockdown of CASC9 increased miR-497-5p expression (Fig. 5e) and elevated miR-497-5p decreased FZD6 expression in BCCs (Fig. 5f). Meanwhile, dual-luciferase reporter assay showed miR-497-5p inhibited the luciferase activity in CASC9-Wt and FZD6-Wt group, with no effect in CASC9-Mut and FZD6-Mut group (Fig. 5g and h). Knockdown of CASC9 decreased the luciferase activity in FZD6-Wt group (Fig. 5i). These results indicated that CASC9 positively regulates FZD6 expression via sponging miR-497-5p in BCCs.

### Knockdown of miR-497-5p reverses tumor growth and metastasis inhibited by silencing CASC9

**Fig. 6:**
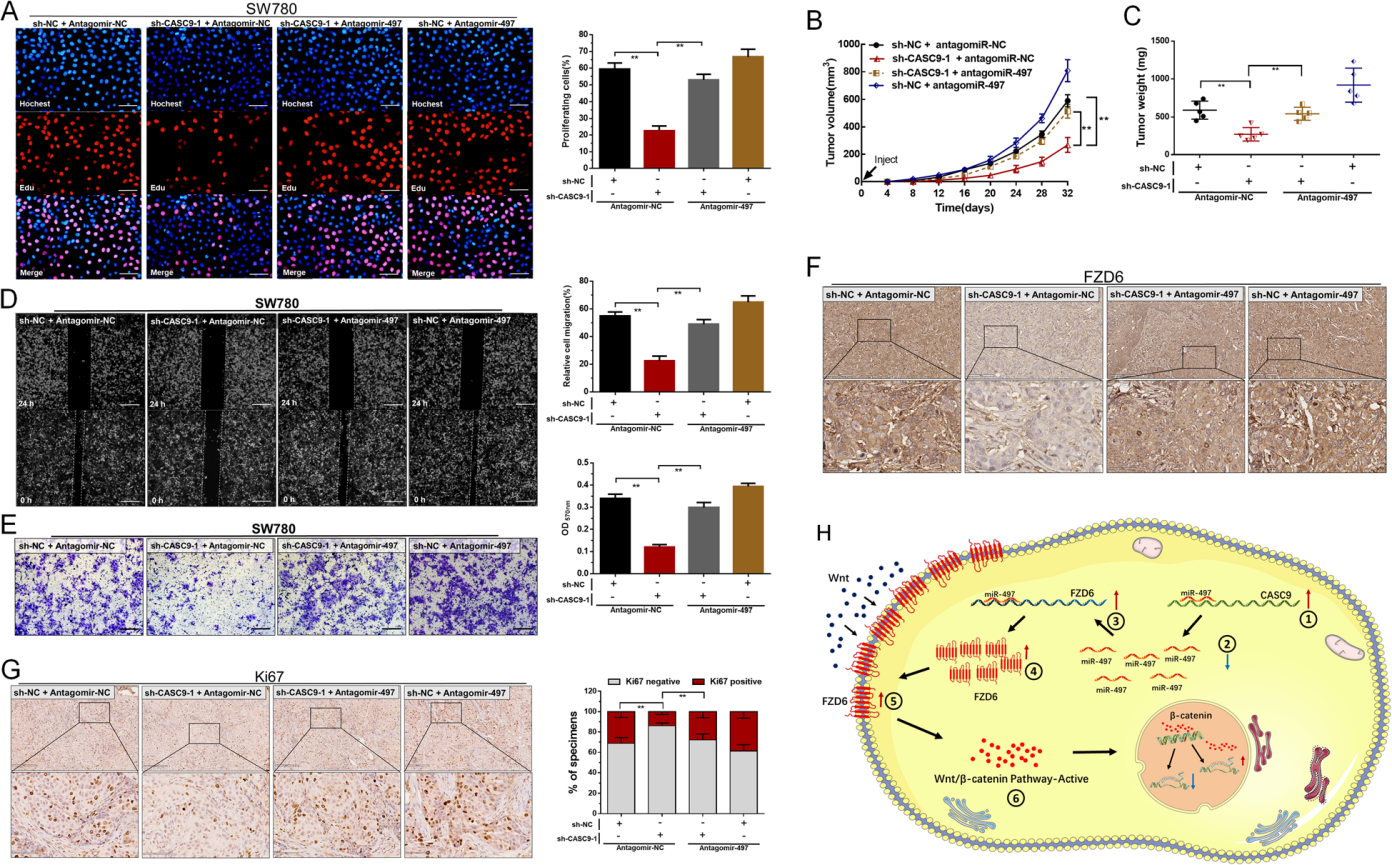
Knockdown of miR-497-5p reverses tumor growth and metastasis inhibition of BCCs induced by silencing CASC9. **a** Knockdown of miR-497-5p significantly reversed growth inhibition of BCCs transfected with shRNA-CASC9 in vitro. **b** and **c** Knockdown of miR-497-5p significantly reversed growth inhibition of BCCs transfected with shRNA-CASC9 in vivo. **d** Knockdown of miR-497-5p significantly reversed migration inhibition of BCCs transfected with shRNA-CASC9 in vitro. **e** Knockdown of miR-497-5p significantly reversed invasion inhibition of BCCs transfected with shRNA-CASC9 in vitro. **f** Knockdown of miR-497-5p significantly reversed FZD6 expression in xenograft transfected with shRNA-CASC9. **g** Knockdown of miR-497-5p significantly reversed Ki67 expression in xenograft transfected with shRNA-CASC9. **h** The schematic diagram of the oncogenic role of CASC9 in BC. The data are shown as the mean ± SD. *P < 0.05; **P < 0.01

To validate the regulatory mechanism of the CASC9/miR-497-5p/FZD6 axis, we further performed miR-497-5p blocking experiments. Our results showed that knockdown of miR-497-5p significantly reversed the proliferation (Fig. 6a), migration (Fig. 6d) and invasion (Fig. 6e) of shRNA-CASC9 group in vitro. Meanwhile, knockdown of miR-497-5p reversed tumor growth of shRNA-CASC9 group (Fig. 6b and c) in vivo. Moreover, knockdown of miR-497-5p significantly reversed FZD6 (Fig. 6f) and Ki67 (Fig. 6g) expression in BCCs. These results indicated that CASC9 promotes malignant phenotypes of BCCs through positively regulating FZD6 expression via miR-497-5p-dependent manner. As shown in Fig. 6h, CASC9 functions as a miRNA sponge to positively regulate FZD6 expression through sponging miR-497-5p and subsequently activates Wnt/β-catenin signaling pathway.

## Discussion

Bladder cancer is the most common and most aggressive malignant primary adult genitourinary tumor [26, 27]. Despite improvements in current clinical treatment such as surgery, radiation therapy, and chemotherapy, the overall survival (OS) time of BC patients has not been significantly improved [28–32]. So novel markers for diagnosis at early stage and more efficient and safer therapeutic method are urgently needed.

The lncRNAs are longer than 200 nucleotides, important members of no-coding RNA family, which have no ability to encode proteins. Recently, the rapid development of RNA genomics has uncovered lncRNAs play key roles in a wide variety of human diseases including cancers, through regulating gene expression at different processing levels, including chromatin modification, transcription and posttranscriptional regulation [33, 34]. For example, LncRNA LNMAT1 could recruit hnRNPL to CCL2 promoter and subsequently epigenetically activate CCL2 expression in BC [12]. LncRNA LUCAT1 could interact with PTBP1 and subsequently facilitate altered alternative splicing of DNA damage related genes in colorectal cancer [35]. LncRNA XIST have also been reported to down-regulate the expression of miR-21 as a miRNA sponge and subsequently increase the expression of IL-12A in primary graft dysfunction [36]. LncRNAs can be distributed in the cytoplasm, nucleus and other cellular substructures, and lncRNAs subcellular localization are closely related to their biology function and molecular roles [37, 38]. CASC9 is a new identified lncRNA located on human chromosome 8 (8q21.11) [39]. Recently, CASC9 originally was identified as a key regulator and the differentially expressed CASC9 have become highlighted in some somatic cancers [25, 40]. However, the clinical significance and biological function of CASC9 in BC are completely unknown.

In the current study, we found CASC9 expression was significantly up-regulated in BC tissues and elevated CASC9 expression was positively correlated with higher histological grade, advanced T stage and poor prognosis. Furthermore, our further experiments demonstrated that CASC9 deletion inhibited tumor growth and metastasis of BC in vitro and in vivo. Mechanistically, through performing comprehensive transcriptional analysis, we found that CASC9 expression was positively correlated with FZD6 expression in BC tissues. Meanwhile, FZD6 expression was also up-regulated in our dataset and knockdown of CASC9 decreased the expression of FZD6. Furthermore, a great number of studies have indicated that FZD6 is a key regulator of Wnt/β-catenin signaling pathway and knockdown of CASC9 decreases FZD6, β-catenin expression and inhibits EMT in BCCs. To explore the regulatory mechanism of CASC9 on FZD6, we detected the subcellular localization of CASC9 using RNA-FISH. These results revealed that CASC9 is distributed mostly in the cytoplasm of BCCs, which suggest that CASC9 may play the regulatory function in posttranscriptional level. Next, through performing bio-information analysis we found CASC9 and FZD6 have potential shared binding sites with miR-497-5p cluster and further experiments demonstrated that CASC9 decreased miR-497-5p expression as a microRNA sponge and subsequently increased FZD6 expression. Moreover, knockdown of miR-497-5p reversed FZD6 expression and malignant phenotypes inhibition of BCCs induced by silencing CASC9.

## Conclusions

In summary, our study revealed that CASC9 positively regulates FZD6 expression through sponging miR-497-5p and subsequently activates Wnt/β-catenin signaling pathway, thus playing an oncogenic role in BC pathogenesis. Based on our results, we propose a network of CASC9/miR-497/FZD6 as an underlying regulatory mechanism mediating occurrence and development of BC. Cumulatively, the findings of the present study suggest that CASC9 is a powerful regulator in BC, which highlight its potential clinical utility as a promising diagnostic and therapeutic target of BC.

## Supplementary information

**Additional file 1: Figure S1.** CASC9 expression is up-regulated in BC. A: The expression of CASC9 in BC was significantly up-regulated in TCGA-BLCA dataset. B: The expression of CASC9 in BC was significantly up-regulated in GSE89006 dataset. C: The CASC9 specific shRNAs significantly decreased CASC9 expression in SW780 and 5637. D: The CASC9 vector significantly increased CASC9 expression in T24. Data are shown as mean ± SD. *P < 0.05; **P < 0.01.

**Additional file 2: Figure S2.** CASC9 promotes cell proliferation and metastasis of BCCs. A: Tumor free mouse proportion of shRNA-CASC9 group was higher than that in the shRNA-NC group. B: The expression of FZD6 and EMT markers in xenografts were determined using qRT-PCR. C: There was no significant difference between the mouse weight of two treatment group. D: The expression of EMT markers in pulmonary metastases were determined using Immunohistochemistry. Data are shown as mean ± SD. *P < 0.05; **P < 0.01.

**Additional file 3: Figure S3.** FZD6 expression is up-regulated in BC. A: FZD6 expression is up-regulated in BC tissues compared with corresponding non-tumor tissues and elevated FZD6 expression is positively correlated with advanced T stage and higher histological grade. B: FZD6 expression is related to the OS and DFS of BC patients in TCGA-BLCA dataset. Data are shown as mean ± SD. *P < 0.05; **P < 0.01.

**Additional file 4: Supplementary Table 1.** Summary of clinicopathological features of tissues of bladder cancer.

**Additional file 5: Supplementary Table 2.** The primer sequences included in this study.

**Additional file 6.** Supplemental Materials and Methods.

## Data Availability

All data in our study are available upon request.

## Abbreviations

lncRNA: Long non-coding RNA
CASC9: Cancer susceptibility candidate 9
FZD6: Frizzled Class Receptor 6
ceRNA: Competing endogenous RNA
BCCs: Bladder cancer cells
OS: Overall survival
DFS: Disease free survival
qRT-PCR: Quantitative real time-PCR
EdU: Ethynyl-2-deoxyuridine
FISH: Fluorescent in situ hybridization
EMT: Epithelial-mesenchymal transition
TCGA: The Cancer Genome Atlas
