# Supplementary figures and images for: Long non-coding RNA CASC9 promotes tumor growth and metastasis via modulating FZD6/Wnt/β-catenin signaling pathway in bladder cancer
Source: J Exp Clin Cancer Res. 2020 Jul 16;39:136. doi: 10.1186/s13046-020-01624-9 (PMC7364562; doi:10.1186/s13046-020-01624-9)

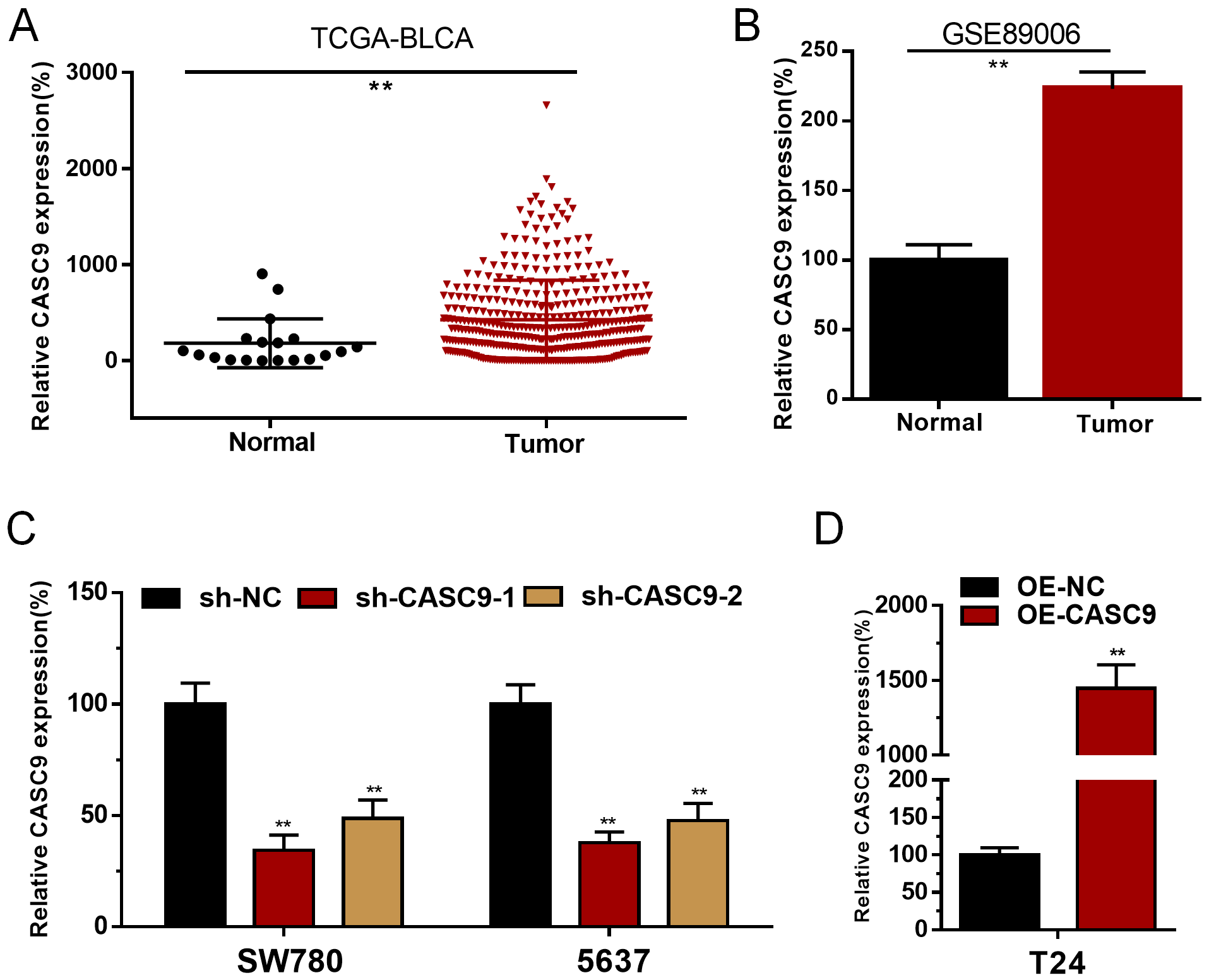

Supplement: Supplementary file 1 — Additional file 1: Figure S1. CASC9 expression is up-regulated in BC. A: The expression of CASC9 in BC was significantly up-regulated in TCGA-BLCA dataset. B: The expression of CASC9 in BC was significantly up-regulated in GSE89006 dataset. C: The CASC9 specific shRNAs significantly decreased CASC9 expression in SW780 and 5637. D: The CASC9 vector significantly increased CASC9 expression in T24. Data are shown as mean ± SD. *P < 0.05; **P < 0.01. [file 13046_2020_1624_MOESM1_ESM.tif]

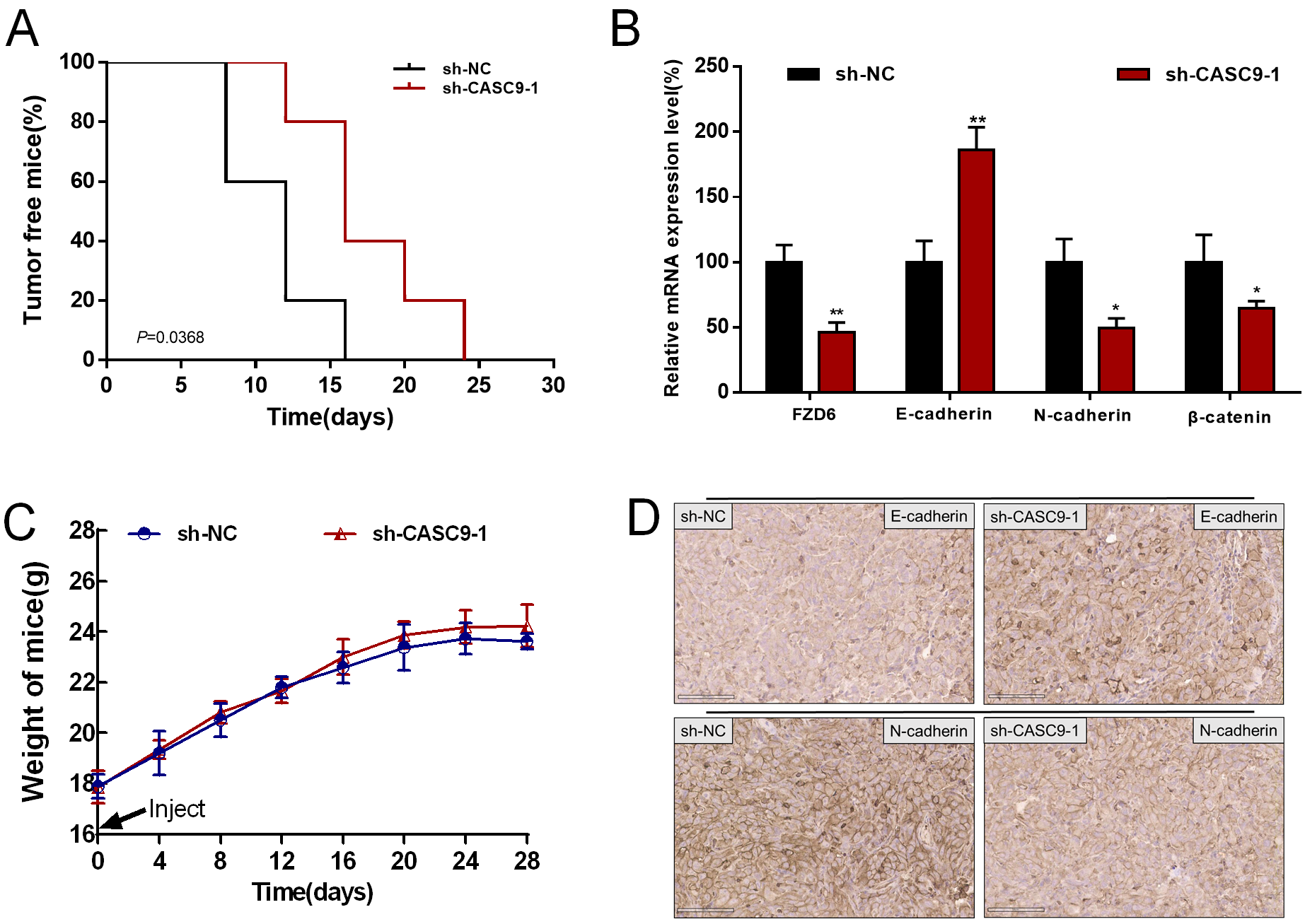

Supplement: Supplementary file 2 — Additional file 2: Figure S2. CASC9 promotes cell proliferation and metastasis of BCCs. A: Tumor free mouse proportion of shRNA-CASC9 group was higher than that in the shRNA-NC group. B: The expression of FZD6 and EMT markers in xenografts were determined using qRT-PCR. C: There was no significant difference between the mouse weight of two treatment group. D: The expression of EMT markers in pulmonary metastases were determined using Immunohistochemistry. Data are shown as mean ± SD. *P < 0.05; **P < 0.01. [file 13046_2020_1624_MOESM2_ESM.tif]

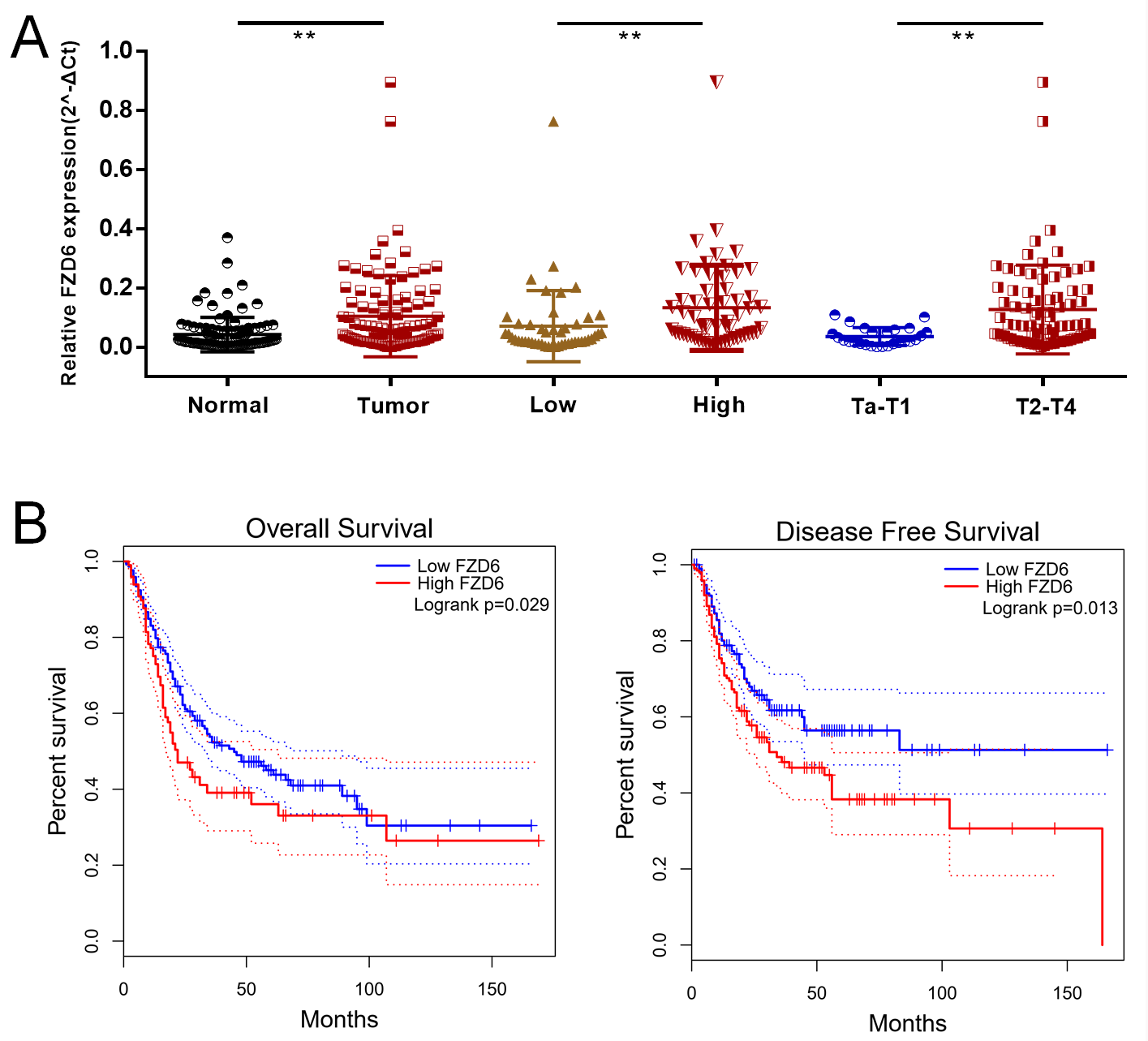

Supplement: Supplementary file 3 — Additional file 3: Figure S3. FZD6 expression is up-regulated in BC. A: FZD6 expression is up-regulated in BC tissues compared with corresponding non-tumor tissues and elevated FZD6 expression is positively correlated with advanced T stage and higher histological grade. B: FZD6 expression is related to the OS and DFS of BC patients in TCGA-BLCA dataset. Data are shown as mean ± SD. *P < 0.05; **P < 0.01. [file 13046_2020_1624_MOESM3_ESM.tif]
