# Supplementary material for: Long non-coding RNA CASC9 promotes tumor growth and metastasis via modulating FZD6/Wnt/β-catenin signaling pathway in bladder cancer
Source: J Exp Clin Cancer Res. 2020 Jul 16;39:136. doi: 10.1186/s13046-020-01624-9 (PMC7364562; doi:10.1186/s13046-020-01624-9)
Supplement: Supplementary file 4 — Additional file 4: Supplementary Table 1. Summary of clinicopathological features of tissues of bladder cancer. [file 13046_2020_1624_MOESM4_ESM.docx]

**Supplementary Table1. Summary of clinicopathological features of tissues of bladder cancer**

| **Pt No.** | **Sex** | **Age** | **Stage** | **Grade** | **Pt No.** | **Sex** | **Age** | **Stage** | **Grade** |
| --- | --- | --- | --- | --- | --- | --- | --- | --- | --- |
| 1 | M | 66 | T2bN0M0 | H | 54 | F | 60 | T2aN0M0 | H |
| 2 | M | 53 | T1N0M0 | L | 55 | M | 68 | T1N0M0 | L |
| 3 | M | 75 | T2bN0M0 | H | 56 | M | 72 | T2bN0M0 | H |
| 4 | F | 64 | T1N0M0 | L | 57 | F | 71 | T1N0M0 | L |
| 5 | M | 58 | T3aN0M0 | H | 58 | M | 54 | T3aN0M0 | H |
| 6 | M | 65 | T2bN0M0 | H | 59 | M | 60 | T4aN2M0 | H |
| 7 | F | 38 | T3aN0M0 | H | 60 | M | 52 | T2aN0M0 | L |
| 8 | M | 59 | T2bN0M0 | H | 61 | F | 67 | T2aN0M0 | L |
| 9 | M | 43 | T3aN0M0 | H | 62 | M | 71 | T1N0M0 | L |
| 10 | F | 64 | T2bN0M0 | H | 63 | F | 67 | T2bN0M0 | L |
| 11 | M | 69 | T1N0M0 | L | 64 | M | 82 | T2aN0M0 | L |
| 12 | M | 72 | T1N0M0 | H | 65 | M | 51 | T4aN0M0 | H |
| 13 | F | 89 | T3aN0M0 | L | 66 | M | 58 | T4aN0M0 | H |
| 14 | M | 68 | T2bN0M0 | H | 67 | M | 49 | T3bN1M0 | H |
| 15 | F | 63 | T3aN0M0 | H | 68 | M | 54 | T1N0M0 | L |
| 16 | M | 63 | T2bN0M0 | H | 69 | M | 79 | T1N0M0 | H |
| 17 | M | 78 | T2aN0M0 | L | 70 | F | 73 | T3aN0M0 | H |
| 18 | M | 70 | T2aN0M0 | L | 71 | M | 69 | T2bN0M0 | L |
| 19 | F | 41 | T2aN0M0 | L | 72 | F | 77 | T1N0M0 | L |
| 20 | M | 59 | T2bN0M0 | H | 73 | M | 65 | T3aN0M0 | L |
| 21 | F | 73 | T2aN0M0 | L | 74 | M | 56 | T1N0M0 | H |
| 22 | M | 67 | T2bN0M0 | H | 75 | F | 63 | T2aN0M0 | L |
| 23 | F | 61 | T3aN0M0 | H | 76 | M | 61 | T4aN2M0 | H |
| 24 | F | 51 | T1N0M0 | L | 77 | M | 49 | T1N0M0 | H |
| 25 | M | 58 | T4aN3M0 | H | 78 | M | 70 | T1N0M0 | L |
| 26 | M | 63 | T2aN0M0 | L | 79 | F | 67 | T2bN0M0 | H |
| 27 | M | 57 | T4aN0M0 | H | 80 | M | 58 | T3aN0M0 | L |
| 28 | M | 54 | T2bN0M0 | H | 81 | M | 63 | T2aN1M0 | H |
| 29 | M | 58 | T4aN0M0 | H | 82 | F | 64 | T3aN0M0 | L |
| 30 | M | 63 | T2aN0M0 | L | 83 | M | 65 | T4aN2M0 | H |
| 31 | M | 50 | T2bN0M0 | H | 84 | M | 61 | T4aN3M0 | L |
| 32 | M | 73 | T3bN0M0 | H | 85 | M | 67 | T2bN0M0 | L |
| 33 | F | 62 | T4aN0M0 | H | 86 | M | 51 | T3aN0M0 | H |
| 34 | M | 41 | T1N0M0 | L | 87 | M | 74 | T2bN1M0 | L |
| 35 | M | 62 | T4aN0M0 | H | 88 | M | 68 | T4aN2M1 | H |
| 36 | M | 76 | T2bN0M0 | L | 89 | F | 75 | T3aN0M0 | L |
| 37 | M | 59 | T4aN0M0 | H | 90 | M | 49 | T3aN0M0 | L |
| 38 | F | 74 | T3aN0M0 | H | 91 | M | 53 | T2aN0M0 | H |
| 39 | F | 70 | T1N0M0 | L | 92 | M | 50 | T2aN0M0 | H |
| 40 | M | 25 | T1N0M0 | L | 93 | M | 83 | T1N0M0 | L |
| 41 | F | 72 | T1N0M0 | L | 94 | M | 60 | T4aN0M0 | H |
| 42 | M | 63 | T3aN0M0 | H | 95 | M | 70 | T3bN2M0 | H |
| 43 | M | 46 | T1N0M0 | L | 96 | M | 36 | T4aN1M0 | L |
| 44 | M | 86 | T1N0M0 | L | 97 | M | 73 | T2aN0M0 | H |
| 45 | M | 70 | T2bN0M0 | H | 98 | M | 65 | T3aN1M0 | H |
| 46 | M | 49 | T1N0M0 | L | 99 | F | 79 | T3aN0M0 | H |
| 47 | M | 61 | T3aN0M0 | H | 100 | F | 34 | T3aN0M0 | H |
| 48 | M | 53 | T2aN0M0 | L | 101 | M | 48 | T1N0M0 | L |
| 49 | M | 73 | T2bN1M0 | H | 102 | M | 81 | T4aN2M0 | L |
| 50 | M | 47 | T2aN0M0 | L | 103 | F | 58 | T1N0M0 | L |
| 51 | M | 77 | T3aN0M0 | H | 104 | M | 79 | T1N0M0 | L |
| 52 | M | 66 | T1N0M0 | L | 105 | M | 63 | T3bN0M0 | H |
| 53 | F | 74 | T2bN0M0 | H | 106 | M | 67 | T4aN0M0 | H |

Pt No. patient number; M male; F female; Grade the World Health Organization 2004 classification; H high; L low; Stage the American Joint Committee on Cancer TNM classification.
