# Supplementary material for: Long non-coding RNA CASC9 promotes tumor growth and metastasis via modulating FZD6/Wnt/β-catenin signaling pathway in bladder cancer
Source: J Exp Clin Cancer Res. 2020 Jul 16;39:136. doi: 10.1186/s13046-020-01624-9 (PMC7364562; doi:10.1186/s13046-020-01624-9)
Supplement: Supplementary file 5 — Additional file 5: Supplementary Table 2. The primer sequences included in this study. [file 13046_2020_1624_MOESM5_ESM.docx]

**Supplementary Table2. The primer sequences included in this study.**

| **Gene** | **Accession number** |  | **Primer sequences(5’-3’)** |
| --- | --- | --- | --- |
| CASC9 | HGNC:48906 | Forward | TTGGTCAGCCACATTCATGGT |
|  |  | Reverse | AGTGCCAATGACTCTCCAGC |
| FZD6 | HGNC:4044 | Forward | AGAGGTGAAAGCGGACGGA |
|  |  | Reverse | AGAGAGTCTGGAGATGGATGCT |
| E-cadherin | HGNC: 1748 | Forward | ATTTTTCCCTCGACACCCGAT |
|  |  | Reverse | TCCCAGGCGTAGACCAAGA |
| N-cadherin | HGNC: 1759 | Forward | AGCCAACCTTAACTGAGGAGT |
|  |  | Reverse | GGCAAGTTGATTGGAGGGATG |
| Vimentin | HGNC: 12692 | Forward | AGTCCACTGAGTACCGGAGAC |
|  |  | Reverse | CATTTCACGCATCTGGCGTTC |
| β-catenin | HGNC: 2514 | Forward | GAATGTCTGAGGACAAGCCACAAG |
|  |  | Reverse | TGGGCACCAATATCAAGTCCAA |
| Slug | HGNC: 11094 | Forward | GCATTTCTTCACTCCGAAGC |
|  |  | Reverse | TGAATTCCATGCTCTTGCAG |
| MMP14 | HGNC: 7160 | Forward | TGCCCAATGGAAAGACCTAC |
|  |  | Reverse | CATCACTGCCCATGAATGAC |
| β-actin | HGNC: 132 | Forward | GCGGACTATGACTTAGTTGCGTTACA |
|  |  | Reverse | TGCTGTCACCTTCACCGTTCCA |
