# Supplementary material for: Long non-coding RNA CASC9 promotes tumor growth and metastasis via modulating FZD6/Wnt/β-catenin signaling pathway in bladder cancer
Source: J Exp Clin Cancer Res. 2020 Jul 16;39:136. doi: 10.1186/s13046-020-01624-9 (PMC7364562; doi:10.1186/s13046-020-01624-9)
Supplement: Supplementary file 6 — Additional file 6. Supplemental Materials and Methods. [file 13046_2020_1624_MOESM6_ESM.docx]

**Supplemental Materials and Methods**

**Data extraction and bioinformatics analysis**

Gene expression profiles was downloaded from The Cancer Genome Atlas (TCGA-BLCA) database and the Gene Expression Omnibus (GSE89006) database. The edgeR package was used to normalize gene expression and analysis differentially expressed lncRNA in TCGA-BLCA detaset. The limma package was used to analysis differentially expressed lncRNA in GEO detaset. The survivalR package was used to analysis prognosis of bladder cancer in TCGA-BLCA detaset.

**Clinical sample collection and cell culture**

Fresh bladder cancer tissue samples and pair-matched normal tissue samples were obtained from patients who underwent radical cystectomy. After resection fresh bladder cancer tissue and pair-matched normal adjacent bladder tissue obtained from the same patient were snap-frozen in liquid nitrogen immediately. All patients included in this study signed informed consent and this study was approved by the Institutional Review Board of Zheng Zhou University First Hospital, Zheng Zhou, China. The normal urothelial cell line and bladder cancer cell lines were purchased from the Institute of Cell Research, Chinese Academy of Sciences, Shanghai, China. Corresponding plates were placed at 37 °C with a humidified atmosphere of 5 % CO2 in incubator.

**RNA extraction and quantitative real-time PCR**

The total RNA of the tissue samples and the transfected cells were extracted using the Trizol reagent (Invitrogen, Carlsbad, CA, USA) according to the manufacturer’s instructions. The detailed primer sequences included in this study are shown in Supplementary Table2. Quantitative real-time PCR was performed using the ABI PRISM 7000 Fluorescent Quantitative PCR System (Applied Biosystems, Foster City, CA, USA) according to the manufacturer’s instructions and normalized to β-actin or U6 small nuclear RNA.

**Western blotting analysis**

Total cell lysates were prepared in a 1× sodium dodecyl sulfate buffer. Total protein was separated by sodium dodecyl sulfate-polyacrylamide gel electrophoresis and transferred onto nitrocellulose membranes. Then the membrane was blocked with 5% non-fat milk and incubated with primary antibodies at 4℃ overnight. After incubation with the primary antibody FZD6/Ki67/Slug/MMP14 (1:2000; Abcam, Hong Kong, China) or E-cadherin/N-cadherin/vimentin (1:2000; Cell Signaling Technology, USA), the blots were incubated with goat anti-rabbit secondary antibody (Abcam, Hong Kong, China) and visualized with enhanced chemiluminescence using an ECL kit (Beyotime Biotechnology, China).

**Cell proliferation assays**

Cell Counting Kit-8 (CCK-8) and ethynyl-2-deoxyuridine (EdU)-incorporation assay were used to determine BC cell proliferation. For the CCK-8 assay, the absorbance was finally determined at a wavelength of 450 nm using a microplate reader (Bio-Rad, Hercules, CA, USA) using CCK-8 (Beyotime Inst Biotech, China) according to the manufacturer’s instructions. For the EdU-incorporation assay, after transfection with the corresponding vector, the cells were incubated with 50 μM EdU (RiboBio, Guangzhou, China) according to the manufacturer’s instructions. Finally, cell fluorescence was visualized using fluorescence microscopy.

**Cell metastasis assays**

Wound-healing assays and transwell assays were used to determine the migratory abilities and invasive abilities of BCCs, respectively. For the wound-healing assay, after transfection with the corresponding vector, the cells were incubated for 24 h; then, a wound was created using a sterile 200-μL pipette tip. Finally, cell migration was monitored under an optical microscope (Olympus, Japan), and the migration distance was calculated by HMIAS-2000. For the transwell assay, 5×10^4^ cells were seeded into the upper chamber with serum-free medium, and medium with 10% FBS was added into the lower chamber. After incubation for 24 h, the cells remaining in the upper chamber were wiped off, and the cells that had migrated to the bottom surface were fixed with 4% paraformaldehyde and imaged.

**RNA fluorescent in situ hybridization (FISH)**

FISH assay was performed using Ribo^TM^ Fluorescent In Situ Hybridization Kit (Ribobio Company, China). CASC9 and U6 probes were designed and synthesized by Ribobio Company and labeled with Cy3 fluorescent dye. RNA FISH were performed using fluorescent in situ hybridization kit (RiboBio) following the manufacturer's instructions. Fluorescence detection was performed with a confocal laser-scanning microscope (Leica, Germany).

**Dual-luciferase reporter assay**

Dual-luciferase reporter assays were performed using a Dual-Luciferase Reporter Assay System (Promega, USA) according to the manufacturer's instructions. Briefly, CASC9-WT/MUT and FZD6-WT/MUT were co-transfected into SW780 with mir-497 agomir/ NC using Lipofectamine 3000 (Invitrogen, USA) and incubated for 48 h. Finally, the luciferase activities were measured using a microplate reader (Bio-Rad, Hercules, CA, USA).

**Mouse model experiments**

All animal experiments were approved by the Institutional Animal Care and Use Committee (IACUC) of The First Affiliated Hospital of Zhengzhou University and Peking University First Hospital (Beijing, China) and conducted in accordance with its recommendations and ethical regulations. For the tumour xenograft implantation experiment, 1×10^6^ SW780 cells were injected subcutaneously into 5-week-old male BALB/c nude mice (Vital River, Beijing, China), which were subsequently sacrificed 5 weeks later. For the metastasis experiment, 1 × 10^5^ 5637-Luc cells were suspended in 200 μL PBS and injected into the lateral tail veins of 5-week-old male B-NDG mice (BIOCYTOGEN, Beijing, China). Four weeks later, mice were anaesthetized with isoflurane and D-luciferin sodium salt (150 mg/kg) was injected intraperitoneally, and cancer cells were detected with an in vivo imaging system, Xenogen IVIS (PerkinElmer, MA, USA). The total flux in photons per second was calculated and reported for each mouse's lung and liver region using Living Image 4.3.1 (PerkinElmer/Caliper).

**Immunohistochemistry and immunofluorescence**

Immunohistochemistry and immunostaining were performed on BCCs and the tissue sections collected from the nude mice. For Immunohistochemistry, sections were incubated with the primary antibodies against FZD6/Ki67 (1:2000; Abcam, Hong Kong, China) and E-cadherin/N-cadherin (1:1000; Cell Signaling Technology, USA) followed by incubation with the appropriate secondary antibody. For immunofluorescence, sections were incubated with the primary antibodies against FZD6 (1:500) and E-cadherin/N-cadherin (1:200) followed by incubation with the appropriate secondary antibody. Finally, the sections were visualized under an optical microscope (Olympus, Japan) or under a fluorescence microscope (Olympus, Japan).

**Statistical analyses**

All experimental data from three independent experiments were analyzed by Student’s t-test or χ2 test and results were expressed as mean ± standard deviation. Kaplan-Meier survival analysis was used to evaluate the cumulative survival probability. The correlation between CASC9 expression and FZD6 mRNA expression in BC was examined using Pearson’s correlation analysis. P-values of less than 0.05 were considered to be statistically significant. All statistical tests were conducted by SPSS version 19.0 software (SPSS Inc. Chicago, IL, USA).
